# Clinical metastatic cancer from initiation to colonization: emerging challenges and breakthroughs

**DOI:** 10.1038/s41388-026-03944-0

**Published:** 2026-08-12

**Authors:** Guanglin Niu, Catharina Hagerling

**Affiliations:** 1 Department of Experimental Medical Science, Lund University, Lund, Sweden; 2 Lund University Cancer Center (LUCC), Lund University, Lund, Sweden

**Keywords:** Metastasis, Cancer microenvironment

## Abstract

Metastatic cancer remains largely incurable and is responsible for the majority of cancer-related deaths. Despite this, therapeutic strategies are still predominantly guided by insights from primary tumors, often overlooking the distinct biological features of clinical metastases. In this Review, we provide an overview of current knowledge on clinical metastatic cancer, spanning dissemination, dormancy, colonization, and therapy resistance. We examine the genomic evolution of metastatic clones, including evidence for both early and late dissemination, as well as the impact of treatment-driven selection. Furthermore, we highlight how organ-specific microenvironments shape metastatic outgrowth and therapeutic response and explore how wound-healing and regenerative programs are co-opted during colonization. Finally, we discuss clinical perspectives on treating metastases and outline how advances in single-cell and spatial multi-omics technologies are transforming our ability to interrogate metastatic disease at high resolution, concluding with future perspectives.

## Introduction

Metastatic cancer remains, with few exceptions, incurable [1, 2]. Improved treatment protocols and new therapies have, however, considerably prolonged the life of many individuals with advanced cancer and an increasing number of individuals are today living with metastatic cancer [3]. In 2025, this heterogeneous patient group, in terms of, e.g., cancer type and received treatments, was expected to include around 700,000 individuals in the US alone [4]. Notably, 30% and 20% of individuals with metastatic melanoma and breast cancer, respectively, are estimated to have lived ≥10 years post metastasis diagnosis [4].

Metastatic cancer diagnosis often comes with a tremendous impact on the quality of life and an experience of uncertainty, with many not having an accurate prognostic awareness [5]. It is associated with physical and psychological symptoms due to the disease itself and associated side effects of various on-and-off treatments [6, 7]. Despite aggressive therapy, individuals with metastatic cancer are often treated without curative intent. Instead, the intention is a prolongation of life. This is contrary to early-stage cancer, which is treated with the ambition to cure the patient. Even so, 30-70%, depending on cancer type, of individuals with metastatic cancer are initially diagnosed with early-stage cancer but later recur [4]. Hence, to improve the clinical outcome, future therapeutics ought to target both covert and overt metastases.

Drug development for treating metastatic cancer is falling behind. Around ninety percent of all drugs fail in clinical trials [8–11]. An approach to guide drug development could be to identify potential drug targets on clinical metastatic tumor cells and non-malignant accessory cells in the tumor microenvironment. Research on clinical metastases have, however, often been hindered by lack of, or too sparse metastatic tumor material and previous technical limitations [12]. This has made credible analysis challenging and often impossible. Fortunately, technological advances, including single-cell and spatial technologies [13–15], allow for unprecedented in-depth investigation of even limited quantities of tumor material.

The focus of this review is to provide an overview of current findings in clinical metastatic cancer, emerging insights, challenges, and potential breakthroughs in the treatment of clinical metastatic cancer.

## The metastatic cascade

Metastasis is the most detrimental stage of tumor progression [1, 2]. It arises through a complex, multistep process involving both tumor-intrinsic alterations and dynamic interactions with the microenvironment [16]. While genetic evolution contributes to tumor progression, increasing evidence suggests that metastatic competence is often driven by phenotypic plasticity and reversible cell state transitions rather than metastasis-specific mutations [17].

### Surviving dissemination and dormancy

**Fig. 1:**
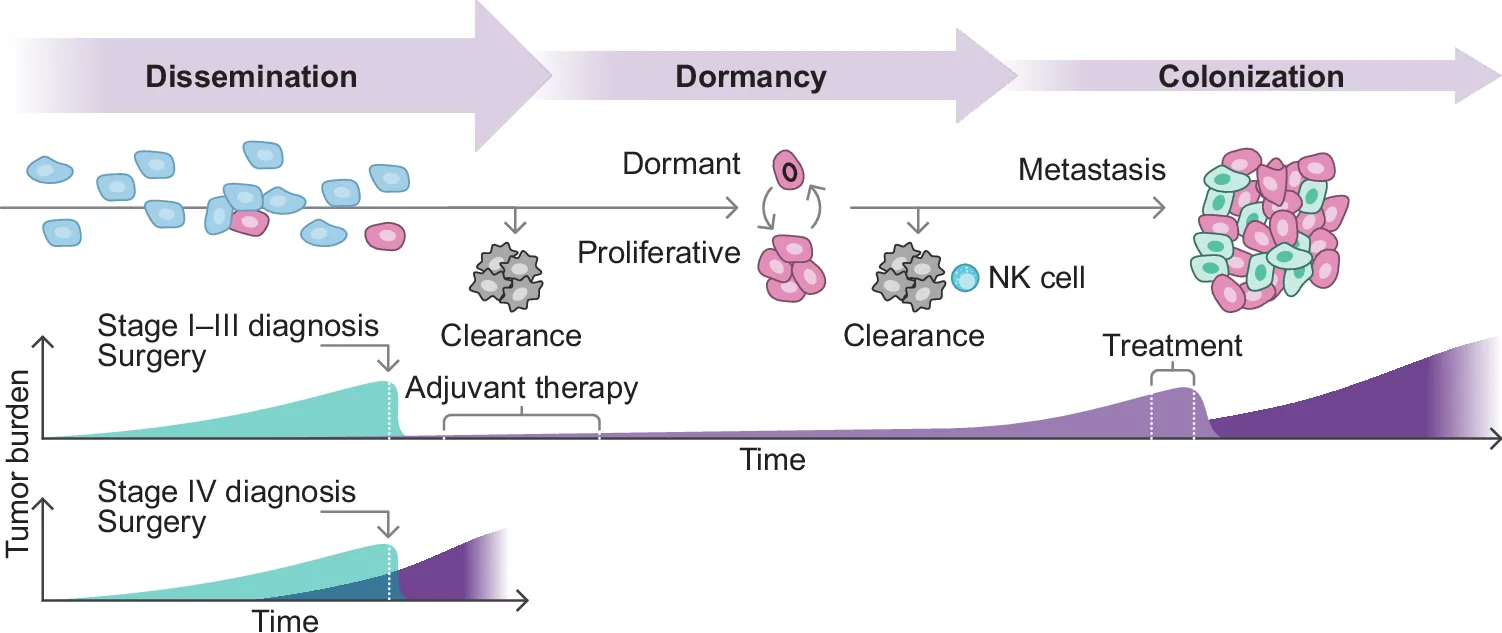
Metastatic cascade. Three phases of the metastatic cascade: Dissemination, Dormancy, and Colonization. Disseminated tumor cells seed multiple organs where they enter a period of dormancy. The time from seeding to metastatic outgrowth can occur months, years and decades after primary tumor resection and treatment. This clinical phenomenom is known as metastatic latency. The figure is adapted from Massagué and Ganesh review1.

The metastatic cascade (Fig. 1) is a highly inefficient process. Only a minority of tumor cells that reach the circulation will form overt metastases [18, 19]. This was demonstrated in a clinical study of cancer patients with peritoneovenous shunts. Patients’ malignant ascites fluid, with millions of tumor cells, was directly routed to the blood circulation via the shunt to reduce abdominal pain and distension. Despite the continuous transfer of millions of tumor cells into circulation for up to 27 months, the majority of the patients who later came to autopsy did not have any large metastatic deposits [20].

Hematogenous dissemination is the main route for distant metastasis formation and the detection of circulating tumor cells (CTCs) in the peripheral blood has a negative prognostic impact for patients with metastatic breast, colorectal and prostate cancer [21–23], (CTCs reviewed in REF [24]). Tumor cells can also spread to discontinuous secondary sites and establish secondary foci by migrating along blood vessels, never entering the lumen [25, 26] or along nerves [27, 28]. Tumor cells can also disseminate through the lymphatics. Until recently, there was a prevailing view in oncology that lymph node metastases gave rise to distant metastases. However, this model has been challenged by work, showing that lymph node metastases and distant metastases develop through different evolutionary trajectories [29, 30]. Importantly, metastasis can also occur through metastasis-to-metastasis dissemination [31, 32] and metastatic tumor cells can home back to the site of the primary tumor [33].

Disseminated tumor cells experience massive attrition due to various stressors in the circulation and once trapped in the capillaries of distant organs [18, 24]. Tumor cells that manage to survive, extravasate, and invade the parenchyma enter a period of dormancy (reviewed in REF [34]). Dormancy can last for an extended period. This explains why recurrence can occur months, years, and even decades after removing the primary tumor. The time from initial tumor cell arrest to overt metastatic outgrowth is known as metastatic latency. Metastatic latency varies between cancer types and is not explained by the distant organ [35]. For instance, breast and lung cancer both metastasize to the brain. However, while lung cancer brain metastases often become clinically detected within a year of primary tumor diagnosis, breast cancer brain metastases can arise decades later [36].

Dormancy is either a non-proliferative cell state—quiescence, or a balance between cell proliferation and cell death due to impaired angiogenesis, or a dynamic equilibrium of persistent tumor growth kept in check by the immune surveillance [37]. Immune cells constitute a significant barrier to metastatic outgrowth [38]. This was evidenced by the accidental transmission of a transplantable organ with latent tumor cells to immunosuppressed recipients that later developed clinical metastases [39, 40]. Natural killer (NK) cells are an example of an immune cell population that can control dormancy [41]. NK cells were reduced in breast cancer liver metastases compared to livers without metastases. Moreover, NK cells correlated inversely to activated hepatic stellate cells and, interestingly, in mouse models, it was observed that NK cells induced a dormant milieu, while activated hepatic stellate cells suppressed NK cells and promoted metastatic outgrowth of breast cancer cells [42].

Besides NK cells, the role of other immune cell populations in regulating the switch from dormancy to metastatic outgrowth remains largely unexplored in clinical metastases. While evidence from primary tumors and experimental models support a role for immune cells in this transition, direct validation in metastatic lesions is still scarce. Notably, infiltration of a specific CD8⁺ T cell subset (CD39⁺PD-1⁺CD8⁺ T cells) in primary breast cancer tumors has been associated with delayed metastatic relapse and shown to maintain metastatic dormancy in mouse models [43]. These observations point to a potential role for specific CD8^+^ T cells in sustaining dormancy in clinical metastases, although this remains to be validated directly in metastatic lesions.

### Colonization and phenotypic plasticity

**Fig. 2:**
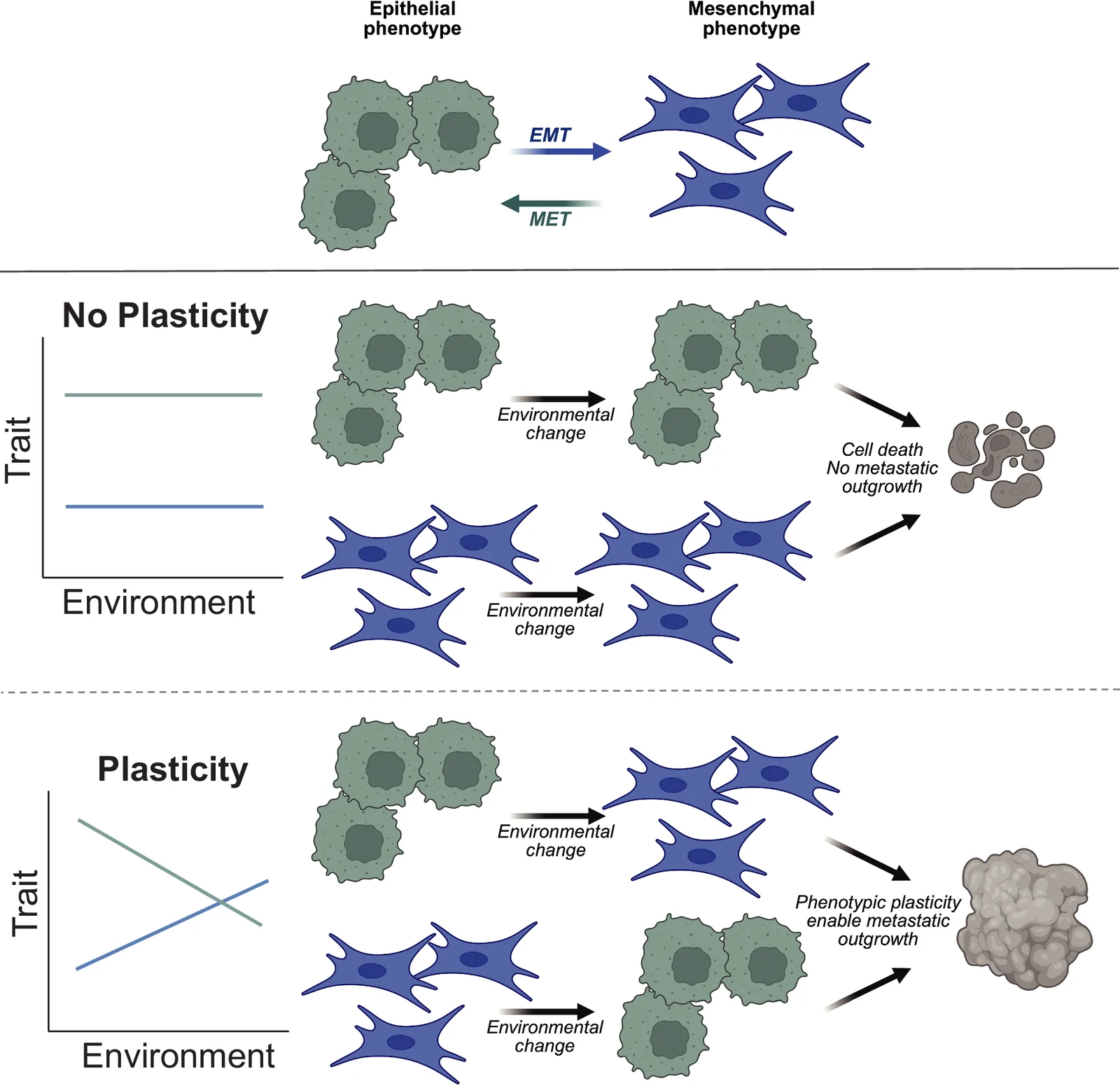
Colonization and phenotypic plasticity. Phenotypic plasticity facilitates the EMT-MET axis and enables metastatic colonization. Metastatic colonization demands adaptive cell-state transitions rather than a fixed phenotype.

Only a minority of the disseminated tumor cells will have the ability to evade immune surveillance, exit dormancy, initiate tenacious proliferation, and form overt metastasis [2, 18, 44]. There is clinical evidence indicating that mesenchymal to epithelial transition (MET) is important for metastatic colonization (reviewed in REF [45]). Breast cancer metastases were found consistently positive for the epithelial marker E-cadherin irrespective of the E-cadherin status in the corresponding primary tumor [46]. In line with those data breast cancer bone metastases expressed E-cadherin despite originating from E-cadherin negative primary tumors [47]. Moreover, the majority of metastases from breast and prostate cancer had increased E-cadherin expression compared to matched primary tumors. Interestingly, for prostate cancer there was also an inverse correlation between the size of the metastatic foci and expression of E-cadherin, implying that E-cadherin might be more important at the beginning of metastatic colonization and that MET is transient and reversible [48]. Consistent with these clinical observations, experimental studies have demonstrated that metastatic tumor cells of epithelial origin undergo reversal of epithelial-mesenchymal transition (EMT), i.e., MET, to reinitiate tumor growth at a distant site [49–51]. Although phenotypic plasticity has not yet been directly demonstrated in clinical metastases, experimental evidence suggests that this reversible EMT-MET process is driven by phenotypic plasticity (Fig. 2), enabling tumor cells to dynamically transition between epithelial and mesenchymal states in response to microenvironmental cues. In colorectal cancer cell lines, a subpopulation of quasi-mesenchymal cells displayed enhanced invasive and metastatic potential, yet gave rise to predominantly epithelial metastases, underscoring the requirement for MET during colonization [52]. Importantly, phenotypic plasticity appears to represent a broader enabling trait for metastatic success beyond facilitating the EMT-MET alone. Bjornberg et al. demonstrated that even within therapy-naïve tumor populations, rare subclones with elevated phenotypic plasticity, characterized by increased transcriptional entropy and open chromatin at EMT and stemness regulators, exhibited superior lung colonization capacity in vivo compared with low-plasticity counterparts. When injected directly into circulation, these high-adaptation-potential cells outcompeted low-plasticity cells by two- to threefold in lung colonization, suggesting that the advantage lies primarily at the colonization step rather than during dissemination from the primary tumor [53]. This is consistent with the notion that metastatic colonization represents a stringent selective bottleneck that demands adaptive cell-state transitions rather than a fixed resistant phenotype.

Experimental models have also shown that metastasis-initiating tumor cells have a stem-cell-like phenotype akin to regenerative stem cells [54, 55], which renders them resistant to NK cell-mediated immune surveillance [56]. In line with those preclinical data, human lung cancer metastases showed greater stem-like character compared to primary tumors, conferring resistance to NK cell mediated killing [56]. Moreover, ∼65% of disseminated tumor cells in the bone marrow of early breast cancer patients demonstrated a cancer stem cell phenotype [57]. One-third of early-stage breast cancer patients were found to have disseminated tumor cells in the bone marrow, however, only about 40% relapsed by the end of the 10-year follow-up period [58]. The experimentally identified dormancy marker, NR2F1, could stratify the dormant tumor cells. Breast cancer patients with NR2F1^high^ disseminated tumor cells in the bone marrow had longer bone metastasis-free periods than those with NR2F1^low^ disseminated tumor cells [59].

Important insights are being made to understand mechanisms controlling the transition from dormancy to metastatic outgrowth. However, what clinical metastatic determinants explain a thriving colonization at a specific organ and how metastases are sustained over time is poorly understood. Notably, these metastasis-determinants should not be mistaken for the plethora of tumor cell and microenvironment-related factors identified in clinical primary tumors, promoting or preventing tumor progression and distant recurrence [60].

### Tumor microenvironment and pre-metastatic niche

Tumor metastasis is not solely a consequence of disseminated tumor cells but is critically shaped by the ability of primary tumors to systemically precondition distant tissues. Tumor-derived factors, including cytokines, growth factors, and extracellular vesicles, can remodel secondary organ microenvironments and establish permissive pre-metastatic niches prior to tumor cell arrival (reviewed in REF [61]). This aligns with the classical “seed and soil” hypothesis, which posits that successful metastasis depends on the compatibility between disseminated tumor cells and a supportive microenvironment [62]. Importantly, the metastatic niche is not a passive recipient but is dynamically shaped through reciprocal interactions between tumor cells and stromal components, including fibroblasts, immune cells, and endothelial cells. These interactions drive extracellular matrix remodeling, immune modulation, and angiogenesis, which further reinforces a pro-tumorigenic milieu (reviewed in REF [38, 63, 64]). Clinical evidence underscores the systemic nature of these processes. An observational study analyzing a broad panel of 92 immuno-oncology proteins in the serum of 136 patients with metastatic breast cancer found that elevated levels of IL-8 was significantly associated with reduced overall survival, a greater number of metastatic sites and the presence of CTC clusters [65]. This association between elevated IL-8 levels and poor prognosis is consistent with previous findings in advanced breast cancer [66]. Moreover, a large-scale retrospective analysis of 1344 patients with advanced cancer, found that elevated serum IL-8 levels correlated with increased intratumoral neutrophil and monocyte infiltration and poorer outcomes following immune-checkpoint inhibitor therapy [67]. Across cancer types, including colorectal and breast cancers, immune infiltration has emerged as an important prognostic factor [68, 69]. In a comprehensive study of resected metastases from 153 colorectal cancer patients, higher infiltration of adaptive immune cells was associated with prolonged survival and improved therapeutic response, whereas non-responding metastases exhibited lower levels of regulatory T-cell (T_reg_) infiltration [68]. Consistent with these findings, T_reg_ infiltration has also been reported as an independent prognostic marker in metastatic breast cancer [70]. A recent study analyzing 100 scRNA-seq samples comprising more than 460,000 cells generated a comprehensive single-cell transcriptomic atlas of liver metastases across multiple cancer types and provided insights into immune niche evolution. The analysis demonstrated that metastatic immune niches evolve dynamically, transitioning from immune-active states characterized by NK cell-mediated immune surveillance and macrophage-driven angiogenesis, toward immunosuppressive states marked by T_reg_ infiltration and immune exclusion [71]. Beyond systemic priming, local microenvironmental cues play a decisive role in determining the fate of disseminated tumor cells. For example, patients with ER^+^ breast cancer without evidence of metastasis had higher levels of TGF-β2 in bone marrow plasma compared to patients with systemic recurrence. In mouse models, it was shown that TGF-β2 derived from mesenchymal-like cells promoted dormancy [72]. Another extrinsic factor that induces dormancy is astrocyte-deposited laminin-211 in the brain. Loss of astrocytic laminins coincided with disseminated tumor cell proliferation in both humans and mice [73]. Together, these findings highlight that metastasis is a systemic and cooperative process governed by bidirectional tumor-stroma interactions, in which both pre-metastatic niche formation, systemic and local microenvironmental signals critically determine metastatic success.

### Wound-healing programs in metastatic colonization

Metastatic colonization and the metastatic microenvironment share notable similarities with physiological wound healing responses [74]. Tissue injury triggers a coordinated repair program involving inflammation, fibroblast activation, ECM remodeling, angiogenesis, and immune cell recruitment, processes that are also prominent in metastatic niches. Experimental studies have demonstrated that ECM deposition and remodeling in distant organs, together with inflammatory myeloid recruitment and stromal activation, are required for metastatic colonization and outgrowth [75–77]. However, direct evidence from clinical metastases remains comparatively limited. Recent insights from osteosarcoma in vivo models, show that disseminated tumor cells induce epithelial injury in the lung, triggering a chronic, non-resolving fibrotic response characterized by excessive ECM deposition and inflammatory cell infiltration that supports metastatic outgrowth. Notably, these findings were recapitulated in clinical osteosarcoma lung metastases, which exhibited similar changes in epithelial composition and phenotype. Importantly, pharmacological inhibition of fibrosis using the kinase inhibitor nintedanib, clinically approved for pulmonary fibrosis, disrupted niche formation and impaired metastatic progression in vivo, demonstrating that wound-healing-like programs are functionally required for metastatic colonization and a potential therapeutic target [78]. Similarly, in colorectal cancer, metastatic initiation has been linked to regenerative programs activated upon tissue injury [55, 79]. L1CAM, which is absent in homeostatic epithelium but induced during tissue regeneration, identifies a subpopulation of tumor cells with high metastatic capacity. L1CAM^high^ tumor cells emerge following loss of epithelial integrity and exhibit stem-like and chemoresistant properties that are essential for metastatic colonization, further supporting the notion that metastatic cells co-opt injury-associated regenerative states to initiate outgrowth at distant sites. Collectively, these findings support a model in which metastatic colonization is driven by the aberrant activation of wound-healing and tissue regeneration programs in distant organs, thereby creating a permissive niche that sustains tumor survival and metastatic outgrowth.

## Clinical metastatic organotropism

**Fig. 3:**
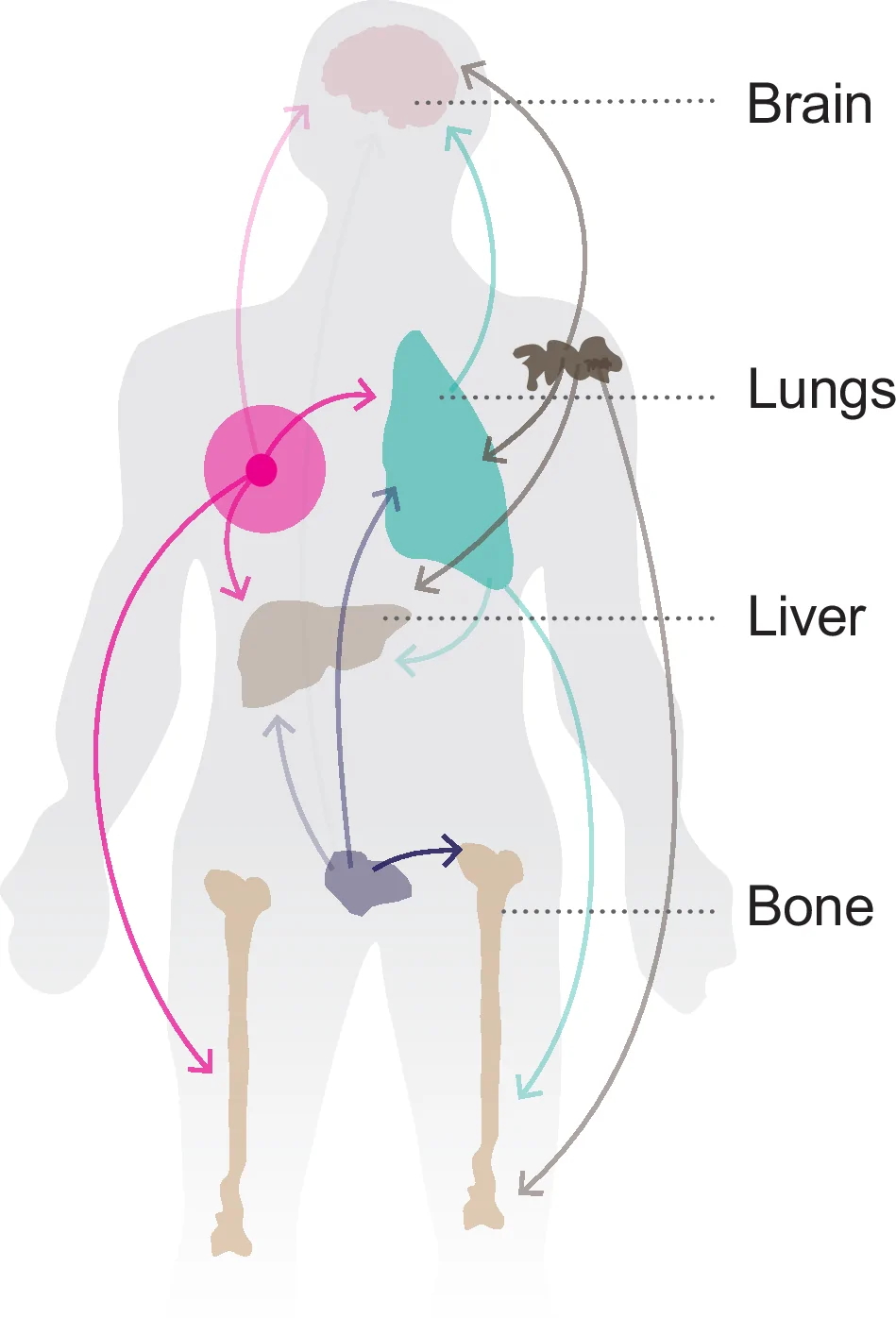
Metastatic behavior of lung cancer, breast cancer, prostate cancer and melanoma. Arrow color intensity indicates relative metastatic frequency.

The most common distant metastatic sites for solid tumors are the lungs, liver, brain, and bone [80]. Notably, despite being highly vascularized, kidneys and spleen are rarely colonized [81]. This suggests that tumor cells lack the cellular capabilities to colonize those organs or that the microenvironment counteracts metastatic outgrowth. The metastatic spread patterns differ among various cancer types and subtypes. However, experimental and clinical evidence indicates that disseminated tumor cells can reach all organs [82, 83], but only in specific organs, depending on the cancer type and subtype, will overt metastatic outgrowth occur [80]. For example, prostate cancer frequently colonizes the bone but rarely forms brain metastases. On the contrary, melanoma often metastasizes to the brain [81] (Fig. 3). Notably, the metastatic site will have an impact on the outcome. Colon cancer patients with liver metastasis have worse outcomes than those with lung metastasis [84]. For breast cancer patients, those with bone metastasis have the best prognosis, while those with brain metastases have the worst [85]. Moreover, the number of metastatic sites can also differ between malignancies. For prostate and ovarian cancer, a single metastatic site is the dominant, while breast and lung cancer often metastasize to multiple organs [86].

Metastatic organotropism can be explained by a combination of circulation patterns, organ-specific microenvironments (reviewed in REF [87–90]), and tumor cells’ intrinsic or acquired capabilities, which determine how fit tumor cells are at adapting and colonizing a particular foreign microenvironment. Metastatic organotropism has been studied in animal models. Experimental observations with serial in vivo selections of cancer cell lines at different metastatic sites have highlighted the role of organ-specific cellular capabilities in metastatic organotropisms [80]. For instance, a human breast cancer cell line, MDA-MB-231, colonizing the bone, brain, and lungs had distinct organ-specific gene expression signatures [91–93]. Whether similar organ-specific metastatic gene signatures exist in humans is still a matter of speculation. However, certain genomic alterations have been associated with metastatic colonization of specific organs. For example, for prostate cancer, bone metastases had a higher frequency of androgen receptor (*AR*) amplification, while lung metastases had a higher frequency of adenomatous polyposis coli (*APC*) and catenin beta-1 (*CTNNB1*) mutations [94]. While this paper shows that metastases at different sites have genomic differences, the potential phenotypic organ-specific metastasis capabilities of these genomic alterations are still largely unexplored. These organ-specific cellular capabilities being crucial in colonization, might hide vulnerabilities and guide organ-specific metastasis drug development. Here, current organ-specific metastasis findings in cancer patients are briefly summarized.

### Lung metastases

Breast cancer and melanoma frequently metastasize to the lungs [81]. For breast cancer, a 6-gene signature was found to be an independent predictor of lung metastasis relapse. The signature was identified by initially comparing the gene expression of five lung metastases with eighteen metastases from other sites and then validated in publicly available transcriptomic data from 721 primary breast tumors [95]. Another study performed transcriptomic and functional enrichment analyses on publicly available clinical data from lung metastases and primary breast, colon, and liver cancer tumors, identifying the cell adhesion molecules pathway as a lung-specific metastasis process [96]. Yet another study analyzed 771 genes in metastases from 176 breast cancer patients. They identified positive regulation of interleukin-6 production and immune response as significantly enriched biological processes in lung metastases compared to metastases at other sites, including bone, brain, and liver [97].

### Liver metastases

The liver is a common metastatic site for breast, pancreatic, and colon cancer [81]. Colon cancer predominantly metastasizes to the liver. This is partly due to the connecting vasculature between the intestinal circulation and the liver [86]. However, in a colorectal cancer cohort with 1652 patients, a fibrotic liver matrix was a powerful predictor of liver metastasis [98]. In experimental pancreatic and colorectal cancer mouse models, liver fibrosis has been shown to be induced by hepatocytes that overexpress serum amyloid A1 and A2. Elevated levels of circulating serum amyloid A1 and A2 were confirmed in pancreatic and non-small cell lung cancer patients with liver metastases compared to patients with only locally advanced cancer [99]. In breast cancer patients, *CLDN2*, encoding the transmembrane cell adhesion protein claudin-2, was significantly upregulated in liver metastases compared to metastases at other sites. Moreover, claudin-2 specifically predicted early liver recurrence [100].

### Brain metastases

More than half of brain metastases are from melanoma, lung, and breast cancer [81]. Brain metastases represent one of the most feared clinical scenarios for cancer patients. Despite aggressive therapies, the median 5-year survival is 2.4% [88], a period associated with an extreme deterioration of quality of life. Recent profiling of tumor cells in non-small cell lung cancer (NSCLC) brain metastases revealed that tumor cells adopt a neural-like cell state, resembling transcriptional programs observed in primary brain tumors. Notably, this cell state was observed exclusively in brain metastases—absent in healthy lung tissue, extracranial metastases or primary tumors. However, tumor cells with a dedifferentiated neural-like cell state were found in primary tumors, raising the question of whether this phenotype drives brain colonization [101]. Together, these findings complement a transcriptional study in breast cancer. Bulk RNA-sequencing of twenty-one primary breast cancer tumors and matched brain metastases identified 3000 differentially expressed genes. The overexpressed genes were interrogated in expression data sets of other metastatic sites, revealing 62 genes upregulated in brain metastases but not in lung, liver, or bone metastases. RET proto-oncogene and human epidermal growth factor 2 (HER2) signaling were proposed as breast cancer brain metastasis drug targets [102].

### Bone metastases

Bone is a common metastatic site for breast and prostate cancer [81]. Bone is typically the first site where breast and prostate cancer metastases are detected [103]. Between 60-85% of breast and prostate cancer patients with advanced cancer harbor bone metastases [104]. Serum levels of soluble intracellular adhesion molecule (sICAM1) and miRNAs are indicators of bone metastasis burden in breast cancer patients and are suggested as bone metastasis biomarkers [105].

Taken together, colonization poses metastasis site-specific cellular capabilities. Further studies are warranted to determine whether they can be therapeutically targetable, or if we can continue to unquestionable treat metastases independent of the distant site. Currently, the great majority of drugs targeting metastatic cancer are systemic rather than considering the distant organ, but organ-specific drugs against metastases could be an opportunity to improve patient survival substantially [1, 106], and distinct tumor-immune microenvironments at various metastatic sites can even co-exist within the same individual [107].

## Genomics of clinical metastatic cancer

### The rise of metastatic clones

**Fig. 4:**
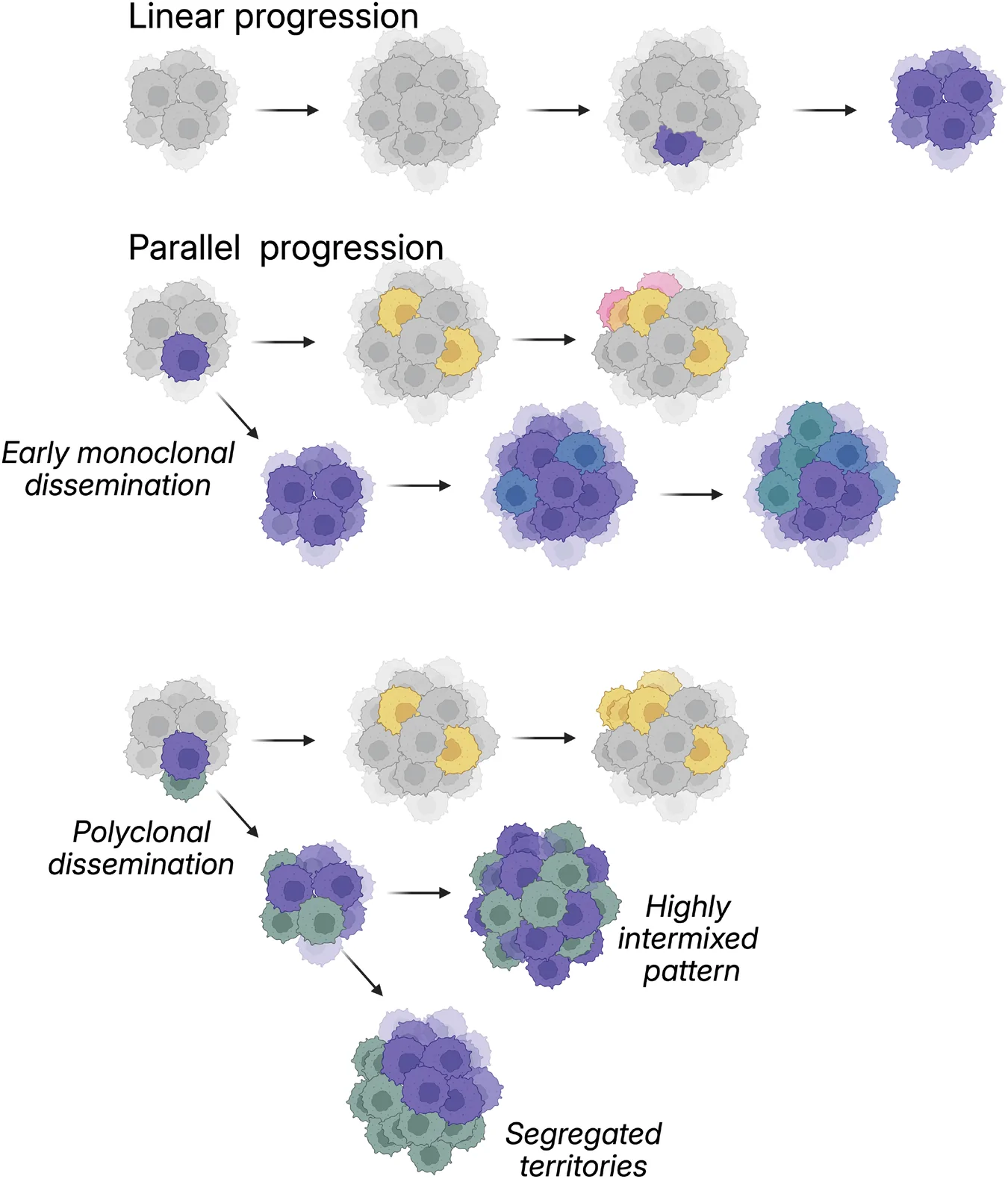
Models of metastatic progression and resulting clonal spatial architectures. In the linear progression model, metastasis arises late, whereas in parallel progression dissemination occurs early. Metastases can be either monoclonal or polyclonal, with polyclonal dissemination giving rise to distinct spatial architectures, including highly intermixed patterns or segregated clonal territories.

There are two models of when tumor cells with metastatic capacity arise and disseminate from the primary tumor (Fig. 4) [108, 109]. One is the prevailing linear model, where a late-arising clone disseminates and gives rise to metastases that, accordingly, will retain a high degree of genetic relatedness to the primary tumor. Indeed, evaluation of the genomic relationship between clinical primary and metastatic samples in, e.g., pancreatic, colorectal, and breast cancer has revealed substantial similarities supporting late dissemination [110–112]. Moreover, the likelihood of developing metastatic cancer is strongly correlated with the size of the primary tumor [113]. On the contrary, in the parallel evolution model, metastases share few genomic alterations with the primary tumor favoring the notion that dissemination occurs early during tumor progression. Genomic and phylogenetic analysis in, e.g., breast, colorectal and lung cancer have found support for early dissemination [114–118]. In NSCLC 25% of the metastases were the result of an early metastatic clone [32]. There is also clinical evidence that dissemination can occur at the premalignant stage. Patients diagnosed with ductal carcinoma in situ, i.e., a breast tumor with malignant cells confined by basement membrane and no signs of invasion, had disseminated tumor cells in the bone marrow [119]; and patients with pancreatic cystic lesions had pancreas epithelial cells in the blood circulation [120]. Moreover, the clinical manifestations of metastases in patients with unknown primary cancer also support early cancer dissemination [121]. In one study including 977 patients with unknown primary cancer and metastases, the primary tumor was only found in 70% of the patients, usually as small, well-differentiated tumors [122].

### Clonality pattern

The continual accumulation of genomic alterations during tumor growth will give rise to intratumor genomic heterogeneity (reviewed in REF [123]) and the eventual formation of clones with metastatic properties [124, 125]. Metastases may arise from a major clone in the primary tumor [117, 126], but they can also arise from a clone not constituting the bulk in the primary tumor. For example, lethal prostate cancer metastases were found arisen from a minor, relatively low-grade cancer foci in the primary tumor [127], and the differential mutation frequency in a breast cancer brain metastasis compared to the matched primary tumor indicated that a minor clone in the primary tumor gave rise to the metastasis [128]. Importantly, multiple metastatic clones can exist in the primary tumor [129], and polyclonal metastatic seeding has been shown for various cancer types. In melanoma, a primary tumor had two subclones with different oncogenic mutations in *CTNNB1*, which both disseminated to the same metastasis [130]. The characterization of multiple prostate cancer metastases from ten patients revealed that half of the patients had polyclonal seeding across different organ sites [31], and 32% of NSCLC metastases had polyclonal seeding, [32]. Interestingly, the clonality pattern differs between metastatic sites, with monoclonality being more frequent in lung metastases than in liver and brain metastases. Monoclonality is also more prevalent in treated metastases compared to untreated metastases [117]. Presumably, due to therapy killing certain clones and selecting for pre-existing drug-resistant clones [123, 131].

A major limitation of genomic analyses of clinical metastases, and our understanding of metastatic evolution, is inadequate sampling. Achieving an accurate representation of the genomic landscape requires comprehensive and spatially resolved sampling strategies [31, 125, 132]. The importance of such approaches was recently highlighted in colorectal cancer, where hundreds of samples from primary tumors and matched synchronous liver metastases were collected from two patients to generate a detailed three-dimensional map of metastatic colonization [133]. This study demonstrates that metastasis is an early and continuous process, driven by the accumulation of multiple clones at metastatic sites. These clones either organize into segregated territories or become highly intermixed (Fig. 4), a pattern that computational modeling implies is driven by low and high cell migration rates, respectively. The authors further suggest that for clinically detectable metastases to arise cooperative colonization of different tumor clones may be necessary and that polyclonality confers an advantage in adapting to new microenvironments and evading immune surveillance, whereas individual clones may face functional constraints. These findings underscore the need for more extensive and systematic sampling of clinical metastases to better define their genomic architecture and to determine whether metastatic outgrowth indeed require polyclonal cooperation. Furthermore, understanding how tumor clones interact and cooperate may reveal novel therapeutic vulnerabilities. Accordingly, future therapeutic strategies may benefit from targeting intra-tumoral clonal dynamics, rather than focusing exclusively on tumor-stroma interactions, as cooperative interactions within the tumor cell compartment may play a critical role in establishing and sustaining macrometastases.

### Genomic alterations

A study by Nguyen et al. performing DNA sequencing of metastases from 25,000 cancer patients concluded, in line with the works of others [134–139], that clinical metastases generally have a higher level of chromosomal instability and higher frequency of whole genome doubling and *TP53* mutations [94]. There are contradicting results regarding whether there are mutations, beyond those required for primary tumor progression, that are drivers of metastasis. Campbell et al. found evidence of potential metastasis driver mutations among rearrangements in metastatic pancreatic cancer. One patient had eight rearrangements that were found in all nineteen metastases, but not in the early-passaged tumor cell line derived from the primary tumor [140]. On the contrary, Ding et al. did not find any metastasis driver mutations in a patient with metastatic breast cancer [128]. Notably, metachronous metastases following treatment harbored more genomic aberrations and driver mutations compared to synchronous metastases. This indicates that treatments change the clonal landscape in favor of drug-resistant clones and that metastasis-specific mutations are interlinked with drug resistance rather than being metastasis driver mutations [117].

Comprehensive genomic profiling of metastases has demonstrated that a substantial proportion of patients harbor genomic alterations that could be targeted therapeutically [141], and genomic panels are increasingly used in clinical practice to guide treatment selection in patients with advanced cancer. Examples include, dabrafenib and trametinib for BRAF V600 mutation positive tumors [142], selpercatinib for *RET* fusions [143], and trastuzumab deruxtecan for HER2-overexpressing solid tumors [144]. However, the clinical benefit of genomically matched therapies compared to standard of care treatments has been inconsistent in randomized trials, often only benefiting a subset of patients [145–148], likely reflecting intrapatient heterogeneity, clonal evolution and limitations in tumor sampling. For example, in the ROME multicenter randomized phase 2 trial, comparing tailored treatment with standard of care, the tailored treatment arm achieved a significantly higher overall response rate (17.5% versus 10% SoC), but unfortunately no improvement in overall survival was observed [146].

## Insights from treating clinical metastatic cancer

Metastatic cancer is most often systemically treated with chemotherapy, targeted therapy, and immunotherapy in a single regime or combination (reviewed in REF [1]). While patients might initially respond to treatment, their metastases will eventually become resistant. Treatment failure is driven, in part, by tumor cell heterogeneity and phenotypic plasticity. In triple negative breast cancer, a CD44^high^ cancer stem cell state has been shown to promote phenotypic plasticity, with androgen receptor (AR) signaling maintaining this state and enabling chemotherapy-induced transitions from CD44^low^ to CD44^high^ cells. Pharmacological inhibition of AR with seviteronel blocked these transitions, reduced metastatic burden, and improved survival in preclinical models. Notably, cytoplasmic AR also emerged as a biomarker of poor prognosis, and patients with high AR expression derived significant benefit from the combination of seviteronel and chemotherapy in a Phase II trial [149]. These findings highlight a clinically strategy to target phenotypic plasticity as a means to prevent the emergence of chemotherapy resistance.

Importantly, different acquired resistance mechanisms have been found in metastases from one patient [150] and multiple resistant subclones existed within the same metastatic lesion [151]. Of note, the most significant genomic alterations found in metastases are associated with various drug-resistance mechanisms [94, 117]. There is not one solution to overcome resistance [152], but to mitigate tumor heterogeneity, upfront combination therapies are preferred to combat metastatic cancer [153–157]. Indeed, combined BRAF and MEK inhibition improved progression-free survival in melanoma patients [158, 159], while sequential treatment did not [160]. Moreover, the prospective study I-PREDICT, showed that patients receiving customized multidrug combinations that targeted a larger fraction of the identified molecular alterations, had significantly better overall survival compared to targeting fewer alterations, emphasizing the importance of personalized combination therapies [161].

Liquid biopsy approaches, particularly the analysis of circulating tumor DNA (ctDNA), have emerged as powerful tools for detecting minimal residual disease and enabling personalized treatment monitoring [162–164]. While circulating tumor cells (CTCs) provide complementary biological insights, ctDNA generally offers superior sensitivity for longitudinal monitoring [164]. In patients with head and neck cancer, ctDNA-guided adjustment of chemoradiotherapy frequency and dosage was associated with an approximately 40% increase in relapse-free survival at three years compared to standard treatment [165]. More broadly, ctDNA has demonstrated high sensitivity for detecting relapse months before radiological evidence [163, 166], raising the possibility of initiating treatment earlier, prior to clinically overt metastatic disease.

The combined 5-year survival for solid metastatic cancer varies between 5% to 30% [167]. The greatest decreases in mortality have been observed in patients with metastatic melanoma and lung cancer [3]. This is primarily due to immunotherapy, which was undeniably a major scientific breakthrough. The introduction of first-generation and second-generation immunotherapies (anti-CTLA4 and anti-PD-1 checkpoint inhibition) and BRAF and MEK inhibitors has [168, 169], for example, doubled the 3-year relative survival for metastatic melanoma over the last decade from approximately 20 to 40% [3]. However, given that only a minority of patients respond, there is a demand for other immunotherapeutic approaches. Notably, organ-specific responses to immunotherapeutics are evident in the clinic, with lung metastases having a better response than brain, bone, and liver metastases [106]. Cancer patients with liver metastases have been observed to have reduced peripheral T-cell numbers. In mouse models, this was explained by liver metastases being infiltrated with immunosuppressive macrophages, which promoted T-cell apoptosis within the liver, resulting in a systemic loss of T-cells [170]. The organ-specific responses to immunotherapy can partially be attributed to the fact that depending on the cancer type and the specific organ, different immune cell populations will contribute to metastatic colonization, (reviewed in REF [171, 172]). Interorgan immune cell heterogeneity is, however, already observed in healthy tissue [173, 174]. For example, while macrophages account for more than 90% of all immune cells in the lung [175], they only comprise 15–20% in the bone [176]. Hence, tailored organ-specific metastasis immunotherapies might have to be applied. Given immune cells’ ubiquity in the human body and their genetic stability compared to tumor cells, understanding how immune cells sustain metastasis at different sites could open new immunotherapeutic avenues.

**Fig. 5:**
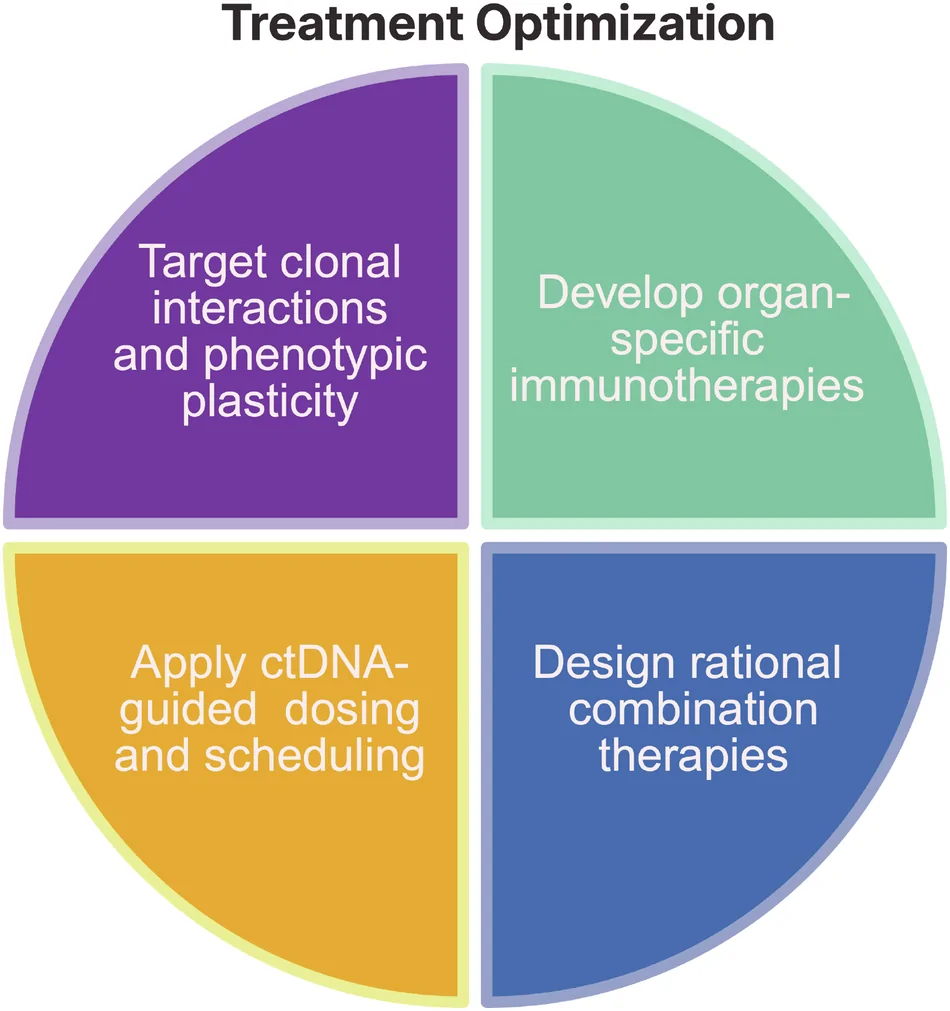
Treatment optimization. Strategies to optimize treatment.

Together, these insights define key strategies for optimizing the treatment of metastatic disease (Fig. 5).

## Challenges and potential breakthroughs

Advancements in diagnostic tools and new therapeutic options have resulted in earlier diagnosis and improved survival for patients with early cancer. However, patients with metastatic cancer still have a woefully poor prognosis. The failure of drug development is argued to be a mismatch between preclinical models and clinical metastases [8, 177]. In vitro models fundamentally differ from the clinical context. While experimental in vivo models have the advantage of recapitulating the complexity of a clinical tumor [177], they often fail to feature crucial components of the human metastatic cascade, such as spontaneous dissemination and an intact immune system [171, 178]. Moreover, treatment paradigms to target metastases are often based on the properties of primary tumors and not the metastases. A more clinically relevant approach to guide treatment and drug development would be to identify potential drug targets on clinical metastasis cancer cells and non-malignant accessory cells in the tumor microenvironment and then proceed with preclinical proof-of-concept experiments.

Until recently, research on clinical metastatic cancer has often been hindered by too sparse metastatic tumor material and previous technical limitations. While advances are being made to investigate clinical distant metastases, the analyzed number of samples is often in the lower range. Moreover, the data is often pooled from lymph node, locoregional, and distant metastases, not corroborating the transcriptomic findings at the protein level and not dissecting tumor cell compartments from the stroma and normal tissue. Although scRNA-seq resolves cellular heterogeneity and has generated a comprehensive overview of various tumor and immune cell populations in clinical metastases [179], the results rarely provide information on their prognostic impact and organ-specificity [180–184]. Moreover, the tissue architecture is destroyed, preventing the discovery of microanatomical niches within tumors and how the catalog of different cells spatially organizes. Also, specific cell populations might be lost in the dissociation process prior to scRNA-seq.

Spatial transcriptomic and proteomic technologies (reviewed in REF [14, 15, 185]) allow for in-depth investigation of clinical metastases, even when limited quantities of tumor material. Spatial profiling of metastases’ diverse tumor compartments could also be a powerful tool to guide combination therapies, which would target different metastatic tumor and immune compartments upfront and thereby hinder therapy resistance. Indeed, diverse immune microenvironments can co-exist within a single tumor [186, 187]. Spatial profiling of primary melanoma revealed the coexistence of both immunoediting and immune evasion in a tumor [186]. Lung cancer brain metastases contain, in line with previous works [188, 189], spatially conserved immune signatures that recur between patients [190, 191], which accordingly could be targets for future immunotherapies. How various microenvironmental niches and cellular interactions in metastases might be therapeutically targeted and impact survival remain largely unknown. Importantly, the immune microenvironment in clinical metastases will have an impact on survival after metastasis diagnosis [70, 192, 193]. Insights from spatial omics will undoubtedly lead to knowledge that can guide new therapeutic strategies to fight metastatic cancer. The emergence of various deep learning models has proven well suited to handle the complexity of spatial omics data [194, 195], and hold further promise for yielding a more comprehensive understanding of metastasis and aid in the repurposing of existing drugs and de-novo metastasis drug development.

## Future perspectives

Although our understanding of clinical metastasis is improving, metastasis remains highly challenging to treat and continues to carry profound consequences for patients. Precision oncology has transformed care in several malignancies; however, genomics-guided treatment has so far benefited only a subset of patients, and durable responses remain limited in many metastatic settings. It is increasingly recognized that combination therapies will most likely play an important role in overcoming tumor heterogeneity and adaptive resistance. At the same time, identifying optimal combinations, sequencing strategies, and patient selection criteria remains a major challenge. Even when patients initially respond to targeted or personalized therapies, these responses are often not durable and rarely curative in advanced disease, suggesting that key aspects of metastatic biology are still not fully understood and inadequately targeted [196].

Metastasis is frequently described as accounting for up to 90% of cancer deaths, although this figure is not strongly supported by primary data. Population-based analyses, such as a Norwegian registry study, suggest that metastases contribute to a majority, but not all, cancer deaths [197]. This highlights an important and still incompletely resolved question: what ultimately drives mortality in patients with advanced cancer? While metastatic burden is clearly central, other factors, including cachexia, systemic inflammation, organ dysfunction, and treatment-related toxicity, likely contribute substantially and may represent underexplored therapeutic targets [198, 199].

In addition to tumor-intrinsic features, host-related factors such as age may influence metastatic behavior (reviewed in REF [200]). In melanoma, clinical observations suggest age-related differences in metastatic patterns, with brain metastases reported more frequently in younger patients [201, 202]. Recent experimental studies propose that these differences may, at least in part, be driven by age-related changes in the stromal microenvironment, involving adipocyte lipid availability, which in turn modulates oxidative phosphorylation in tumor cells [203]. While these findings are intriguing, their direct clinical relevance and generalizability across cancer types remain to be fully established. Similarly, sex has emerged as a potential modifier of metastatic risk, with large registry-based analyses indicating that male sex is associated with a higher risk for distant metastasis and worse overall survival compared to females for a majority of cancer types [204, 205]. Notably, the male sex effects were greater in patients younger than 65 years [204], further suggesting an interaction between age and host factors in shaping metastatic progression. Taken together, these findings suggest that metastasis should be viewed not only as a tumor cell-intrinsic process, but also as one shaped by a broader host context, including systemic metabolism, immunity, age, and sex. This raises the possibility that factors not traditionally considered central to metastasis may nonetheless influence both disease progression and treatment response.

An additional perspective is to consider why overt metastases do not develop, despite the presence of an aggressively growing primary tumor and disseminated tumor cells. While much research has focused on mechanisms that promote metastatic spread, there is growing interest in primary tumor-derived factors that may restrain metastatic outgrowth. This concept, sometimes referred to as concomitant tumor resistance, describes the ability of a primary tumor to suppress metastatic colonization [206]. Preclinical studies have shown that primary tumors can induce systemic immune responses capable of limiting metastatic colonization [207, 208], and may also contribute to maintaining disseminated tumor cells in a dormant state [209, 210]. Some clinical observations have reported the emergence or accelerated growth of metastases following removal of the primary tumor [206], consistent with the loss of such a systemic restraint. However, these observations should be interpreted with caution. The apparent outgrowth of metastases after primary tumor removal may instead reflect surgery-induced systemic changes, including inflammation, and wound-healing responses, which have been shown to promote metastatic outgrowth [211, 212]. Thus, while concomitant tumor resistance provides a compelling framework to explain the absence or delayed emergence of metastases, its relative contribution compared to perioperative effects and other systemic influences remains to be determined.

Overall, metastasis represents a complex biological process occurring within an equally complex systemic environment. While genetic alterations remain central to our understanding, it is increasingly apparent that non-genetic host factors also play important roles in determining metastatic progression and therapeutic response. A deeper understanding of these interactions, and of why metastasis fails to occur in some patients, may open new avenues for more effective and durable treatment strategies.
